# Prothrombotic States in Transcatheter Heart Valve Leaflet Thrombosis (PROSTHESIS): Rationale and Early Results of the Observational Cohort Study

**DOI:** 10.3390/jcdd12020062

**Published:** 2025-02-06

**Authors:** Kajetan Grodecki, Katarzyna Pawlak, Matylda Grodecka, Bartosz Rymuza, Piotr Scislo, Olga Ciepiela, Janusz Kochman, Zenon Huczek

**Affiliations:** 1 1st Department of Cardiology, Medical University of Warsaw, 02-097 Warsaw, Poland; 2 Department of Laboratory Medicine, Medical University of Warsaw, 02-097 Warsaw, Poland; 3 Faculty of Medicine, Wroclaw Medical University, 50-425 Wroclaw, Poland

**Keywords:** transcatheter aortic valve replacement, hypoattenuating leaflet thickening, computed tomography angiography

## Abstract

Subclinical leaflet thrombosis is an imaging phenomenon observed after transcatheter aortic valve implantation (TAVI) and characterized by hypoattenuating leaflet thickening (HALT) on computed tomography angiography. The clinical implications and underlying causes remain uncertain. Hypercoagulability, a component of Virchow’s triad, may contribute to thrombus formation on bioprosthetic leaflets, but data on hypercoagulable disorders in TAVI patients and their impact on HALT are limited. The PROSTHESIS study (Prothrombotic States in Transcatheter Heart Valve Subclinical Leaflet Thrombosis) is a single-center observational cohort study aiming to include 130 TAVI patients. This pilot study aimed to (i) assess the effect of hypercoagulable disorders on HALT prevalence and (ii) evaluate their impact on the natural history of HALT. Patients were screened for common hypercoagulable disorders using genetic and functional assays and underwent multimodal imaging one year after TAVI to detect HALT. In patients with HALT, post-implant imaging was repeated after three months to assess its progression. Early results comparing 52 TAVI patients with 52 matched controls undergoing coronary angiography showed similar thrombophilia prevalence between the groups (16% vs. 12%, *p* = 0.565). HALT occurred in 15% of TAVI patients, more extensively in those with thrombophilia (712 mm^3^ vs. 135 mm^3^, *p* = 0.036). However, thrombophilia was not an independent predictor of HALT. One-year follow-up CTA revealed spontaneous HALT resolution in 63%, stability in 25%, and progression in 12%. This study aims to provide insights into HALT and its mechanisms, which may help prevent complications and improve bioprosthesis durability.

## 1. Introduction

Transcatheter aortic valve implantation (TAVI) has become a treatment option for patients with symptomatic severe aortic stenosis who are at high or extreme surgical risk. Moreover, the transcatheter approach has been recently approved as an alternative to surgical valve replacement in the final, low-risk population [1,2]. As the number of procedures is rapidly increasing worldwide, subclinical leaflet thrombosis in both transcatheter and surgical aortic bioprostheses has raised numerous concerns [3].

Subclinical leaflet thrombosis is defined as hypoattenuating leaflet thickening (HALT) with or without significantly reduced leaflet motion and is diagnosed with multidetector computed tomography (CT) [4]. The prevalence of HALT ranges from 7% to 40%, with the largest registries demonstrating its presence in approximately 13% of patients [3,5,6,7,8,9]. The onset of HALT might occur as early as 2 days after the implantation of a bioprosthesis, and the incidence will increase over time [9]. The natural history of HALT is not well understood, and it is hypothesized that it might potentially progress into clinical leaflet thrombosis [10]. Nevertheless, a small study analyzing two consecutive four-dimensional volume-rendered CT scans showed HALT stability in 73.8% of cases during a 3-/6-month follow-up, while progression was observed in 15.5%, with no patient developing symptoms of valve dysfunction. Interestingly, the occurrence of HALT, as well as its progression, was less likely in patients receiving oral anticoagulation [11].

The clinical consequences of HALT remain uncertain. Early HALT has not been found to be a predictor of mortality, strokes, or transient ischemic attacks [7,12]. However, reduced leaflet motion in HALT has shown a stronger association with cerebrovascular events than in HALT, which does not affect leaflet mobility [12]. Both migration of leaflet thrombus and de novo thrombus formation on severe immobile leaflets may contribute to adverse cerebrovascular complications in the setting of HALT, but further research is warranted to discover its pathophysiological details. More studies are needed also to determine the link between HALT and valve degeneration [5,6,7,8,9].

Virchow’s triad underlies the formation of a thrombus on the surface of a transcatheter heart valve leaflet [4]. Firstly, forces affecting transcatheter heart valves during crimping and deployment may damage its leaflets, thereby increasing their thrombogenicity. Secondly, low-flow states and the post-implant deformation of the transcatheter heart valve might promote HALT through the alternation of hemodynamic flow patterns across the prosthesis [13,14]. Finally, prothrombotic states may impact blood rheology and contribute to the formation of thrombi. The compounds of the Virchow triad can result from patient comorbidities, such as advanced age, chronic kidney disease, diabetes, smoking, or obesity [15]. However, there is a wide spectrum of primary hypercoagulable disorders that have not been studied in the context of HALT. To date, only one study has reported their incidence in analyzed cohorts, but no details regarding the approach to laboratory testing were revealed [5]. Hence, there is a huge gap in knowledge, especially crucial for transcatheter procedures, where the native cusps are not removed and may expose tissue factors to the blood, activating the extrinsic coagulation pathway.

Since very few data are available on the prothrombotic states in TAVI patients and their impact on HALT, we designed a prospective single-center observational cohort study to evaluate whether hypercoagulable disorders affect the formation and natural history of HALT.

## 2. Materials and Methods

### 2.1. Study Design

This study was a prospective observational cohort study. It included patients referred for TAVI due to severe aortic stenosis who had previously been qualified for this type of treatment by the local Heart Team and were not receiving oral anticoagulation.

### 2.2. Aims of the Study

The study aimed to (1) determine the prevalence of hypercoagulable disorders in the population of patients undergoing TAVI; (2) assess the effect of hypercoagulable disorders on the occurrence, characteristics, and natural history of HALT; and (3) evaluate the impact of hypercoagulable disorders on cerebrovascular events in the setting of HALT.

### 2.3. Overview

**Figure 1:**
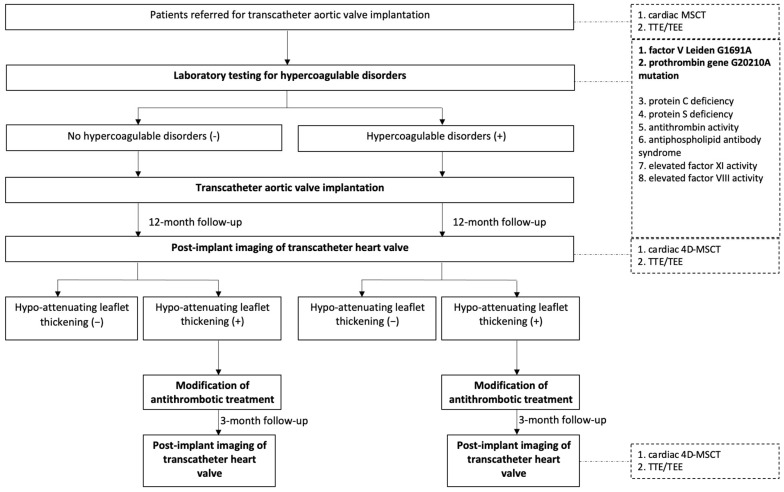
Study design.

In the first stage of the study (hospitalization during the preoperative period), cardiac CT angiography and transthoracic/transesophageal echocardiography were performed in all patients included in the study as part of standard care. Additionally, 10 mL of venous blood was collected for laboratory assessment of hypercoagulable disorders. Based on the test results, patients were divided into two groups: those with thrombophilia and those without thrombophilia (Figure 1). In the next stage of the study, 12 months after TAVI, multimodal post-implant imaging of the transcatheter aortic valve was conducted. For patients with HALT, antithrombotic treatment was modified at the physician’s discretion. Repeated post-implant CT imaging was performed after 3 months to evaluate the progression or resolution of HALT. Patient recruitment began in November 2019 and is expected to be completed within 5 years.

### 2.4. Participant Inclusion Criteria

The inclusion criteria were as follows: (1) severe aortic stenosis (aortic valve area <1.0 cm^2^ and a mean pressure gradient ≥40 mm Hg or a peak jet velocity >4 m/s); (2) qualification for transcatheter aortic valve replacement by the local Heart Team; (3) men and women aged ≥18 years; and (4) voluntary participation with signed informed consent provided [16].

### 2.5. Participant Exclusion Criteria

The exclusion criteria were as follows: (1) transcatheter aortic valve-in-valve implantation; (2) known allergy to contrast media; and (3) contraindications for CT angiography.

### 2.6. Endpoints

**Figure 2:**
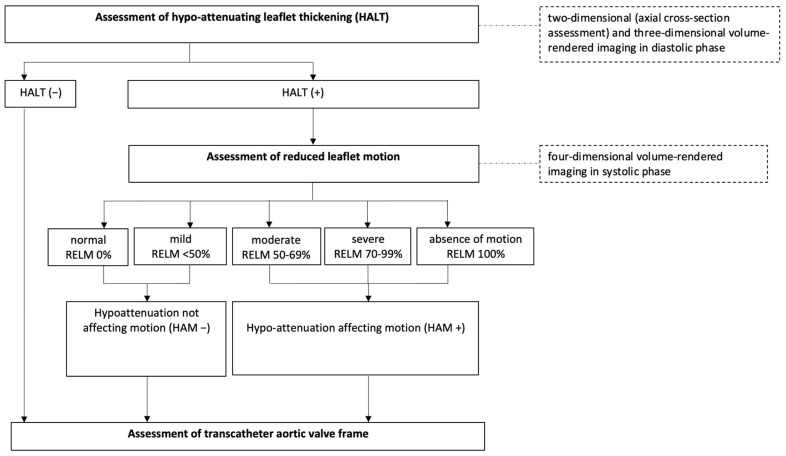
Evaluation of HALT from computed tomography angiography.

The primary outcome was visually identified HALT as the hallmark of subclinical leaflet thrombosis. The hypoattenuating lesions involved the periphery and base of the leaflet and extended to varying degrees to the edges of the leaflet in the center of the bioprosthetic frame. If HALT was identified, a careful assessment of leaflet motion was conducted using 4-dimensional CT imaging. Motion reduction of each leaflet was evaluated using multiphase volume-rendered enface cine projection. Hypoattenuation affecting motion (HAM) was defined as a >50% reduction in leaflet motion relative to the radius of the bioprosthetic frame (Figure 2).

### 2.7. Multislice Computed Tomography

CT scans were performed in all patients at least 48 h before the planned TAVI as part of standard care and assessed with dedicated software (3mensio Structural Heart 10.0; 3mensio Medical Imaging BV, Bilthoven, The Netherlands). CT imaging was conducted during post-discharge follow-up visits 12 months after TAVI to evaluate the transcatheter heart valve and was repeated after 3 months in patients diagnosed with HALT to assess its evolution over time. CT scans were obtained using the standardized protocol described previously [4].

**Figure 3:**
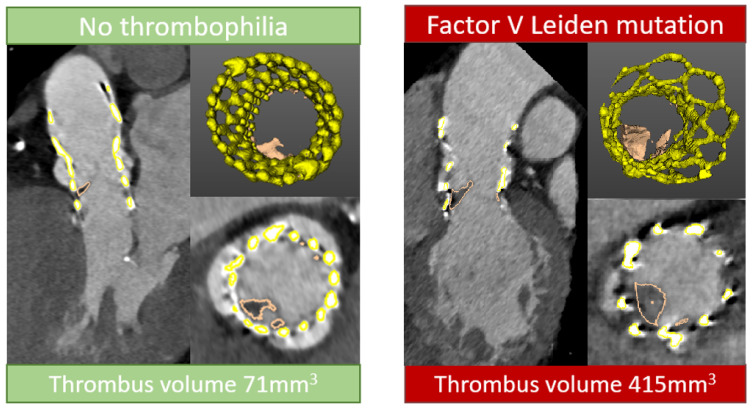
Quantitative thrombus characterization from computed tomography angiography.

Quantitative analysis of HALT was performed using semi-automated software (ValveQuant module, Autoplaque version 2.5; Cedars-Sinai Medical Center, Los Angeles, CA, USA) (Figure 3). The region of interest was manually defined from the base to the top of the bioprosthetic aortic valve. Then, serial multiplanar reformatted images orthogonal to the longitudinal axis of the ascending aorta were rendered to obtain cross-sectional images of the region in question with contouring, comprising 15–20 adjustable points within the inner margin of the stent frame. Leaflet thrombus was defined as voxels between −200 HU and 200 HU within the inner margin of the bioprosthetic frame. The quantitative thrombus parameter was volume (expressed in mm^3^) [17,18]. The software offers excellent reproducibility for HALT volume (Supplementary Materials).

### 2.8. Hypercoagulability Panel

Blood samples were collected at least 24 h prior to TAVI using a winged blood collection set. A discard tube was drawn first to account for the dead space of the tubing and prevent underfilling of the evacuated tube. Subsequently, a venous blood sample of 10 mL was collected into two tubes: (1) one containing 3.2% buffered sodium citrate and (2) one containing tripotassium ethylenediaminetetraacetic acid (K3-EDTA). Standard blood tests were performed on-site in a local hospital laboratory according to the established protocol. The remaining volume of unused samples was frozen at −20 °C and then transferred to −80 °C for storage. Comprehensive hypercoagulability testing was performed in a specialized laboratory. Genetic testing to detect the G20210A Factor II (prothrombin) and G1691A Factor V Leiden mutations was performed with PCR-based methods. Antithrombin and factors VIII and XI activity were measured by aPTT-based clotting assay. The activity of protein C was measured using a chromogenic assay, and activated protein C resistance was functionally tested with a generation assay. Antigenic levels of free protein S were measured using monoclonal antibody-based enzyme immunoassay. Confirmation of LA was based on consensus criteria from the International Society for Thrombosis and Haemostasis [19]. Thrombophilia was defined by the presence of at least one abnormality in the hypercoagulability panel described above.

### 2.9. TAVI and Antithrombotic Regimens

All patients were referred for TAVI after a detailed evaluation by the local Heart Team. The access site (transfemoral, transcarotid, subclavian, or any other) and type of valve (self-expanding or balloon-expandable) were determined at the discretion of the operator. The procedures were performed in hybrid operating rooms under local anesthesia with cautious sedation. The vascular closure technique was decided on-site. Regarding periprocedural pharmacological treatment, patients not on anticoagulation were given loading doses of 300 mg of aspirin and clopidogrel within 24 h before TAVI, and these were continued with 75 mg daily after the procedure. Oral antiplatelet drugs were continued throughout the hospitalization unless major bleeding occurred. Unfractionated heparin was administered as a 5000 IU intravenous bolus immediately after access to the delivery system was obtained, with additional boluses given if the activated clotting time value was below 250 s. Protamine sulfate was administered immediately after successful implantation of the bioprosthesis, at the discretion of the operator, as 1 mg per 100 IU of unfractionated heparin. The antithrombotic regimen at discharge was decided by the on-site physician in accordance with the current practice guidelines of the European Society of Cardiology. In patients with the presence of HALT, antithrombotic treatment was modified at the discretion of the physician.

### 2.10. Follow-Up and Duration of the Study

After discharge from the hospital, each patient had an outpatient clinical visit scheduled for evaluation one year after the TAVI procedure. An additional outpatient clinical visit was scheduled for patients with HALT 3 months after diagnosis. Patients were contacted via telephone every 6 months to assess for adverse events (especially cerebrovascular events) for at least two years after the procedure. Patient recruitment began in November 2019 and was expected to be completed within 5 years. Patients presenting with clinical valve thrombosis underwent examination according to the protocol at the time of hospital admission.

### 2.11. Ethical Issues

The PROSTHESIS study was approved by the Bioethics Commission at the Medical University of Warsaw (KB/128/2018). Recording of adverse events was conducted according to good clinical practice, the ethical principles described in the Declaration of Helsinki, the requirements of the European Medicines Agency, and local legal and regulatory requirements. Data storage was performed in accordance with local data protection laws. Competent authorities and sponsor-authorized persons could request access to trial documentation in case of an inspection or audit. Direct access to these documents was guaranteed by the principal investigator. Documentation could be copied during inspection or audit if the identity of the participant had been made unrecognizable.

### 2.12. Statistical Analysis

To assess the normality of distribution, the Shapiro–Wilk test was used. Continuous and categorical data were presented as means ± SDs or medians (interquartile ranges [IQRs]) and frequencies (percentages), respectively. Comparison of continuous variables was performed using the Student’s *t*-test or the Mann–Whitney U test, and for categorical variables, a comparison was made using either the chi-squared or Fisher exact tests. To determine the risk factors of the primary endpoint, univariate logistic regression analysis was performed. Multivariable logistic regression for early results was adjusted for age and gender due to a limited number of events. Multivariable logistic regression models were built with covariates that had an associated *p*-value < 0.10 in the univariable model in the final study [20,21]. Time to adverse clinical outcomes (such as stroke/transient ischemic attack) was estimated using the Kaplan–Meier technique and compared by the stratified log-rank test. A *p*-value <0.05 was considered statistically significant. Analyses were performed using SPSS version 23.0 software (SPSS Inc., Chicago, IL, USA).

## 3. Early Results

To compare the prevalence of thrombophilia in patients with AS and the general population, we enrolled 52 patients undergoing TAVI for severe AS and matched them with 52 patients admitted for elective invasive coronary angiography due to suspected stable coronary artery disease. There were no differences between the groups in clinical characteristics (Supplementary Table S1).

The prevalence of any thrombophilia was comparable between the TAVI and control groups (16% vs. 12%, *p* = 0.565). Similarly, there were no differences in the distribution of thrombophilia factors between these groups (Table 1). All patients with Factor V Leiden and Prothrombin G20210A mutations were heterozygous. The single patient with antiphospholipid syndrome was double-positive.

There were no differences in clinical characteristics between patients with and without thrombophilia in the TAVI group (Table 2). The single patient with a history of deep vein thrombosis in the TAVI group had been diagnosed with Factor V Leiden.

HALT was detected one year after TAVI in eight patients (15%), all with self-expandable valves, and was not more prevalent in patients with thrombophilia factor (38% vs. 11%, *p* = 0.095). Of three HALT patients with thrombophilia, one had factor V Leiden mutation, one had prothrombin G20210A mutation, and one had antithrombin deficiency. Notably, patients with thrombophilia presented more extensive HALT, as all three (100%) incidences of RELM were noted in this group. This observation was further confirmed with a quantitative assessment of HALT, which showed a higher thrombus volume in patients with thrombophilia as compared to those without (712 mm^3^ [IQR 445–712 mm^3^] vs. 135 mm^3^ [IQR 111–374 mm^3^], *p* = 0.036). Thrombophilia was not an independent predictor for HALT in an exploratory multivariable logistic regression (OR 3.3 [IQR 0.5–20.3] *p* = 0.183) adjusted for age and gender.

None of the patients presented elevated transvalvular gradients in echocardiographic examination at one year. Therefore, it was decided not to start oral anticoagulation in HALT patients. In the follow-up CTA, spontaneous resolution of HALT was observed in five (63%) patients (one with and four without thrombophilia), and no change in HALT was observed in two (25%) patients (one with and one without thrombophilia). Finally, a progression of HALT was noted in one (12%) patient with Prothrombin G20210A mutation.

## 4. Discussion

To our knowledge, PROSTHESIS is the first study to prospectively evaluate the role of hypercoagulable disorders in HALT. With our early results, we have demonstrated that (1) the prevalence of thrombophilia in the TAVI population is comparable to that in the general population, (2) thrombophilia may not be associated with increased incidence of HALT, and (3) HALT observed in patients with thrombophilia might be more severe and less prone to spontaneous resolution.

The prevalence of thrombophilia varies among different populations; however, its estimated incidence among Caucasians is ~10%. The most prevalent thrombophilia is due to factor V Leiden (3–7%) and prothrombin G20210A mutation (1–3%), while remaining defects, such as protein C and S deficiencies, are significantly less common (0.1–0.5%) [22,23]. Our study showed that the distribution of thrombophilias in the TAVI population is comparable to that in the general population, as described in the literature [24]. Our study further confirmed this by matching patients undergoing TAVI with patients admitted for invasive diagnostics of coronary artery disease.

The low incidence of thromboembolic events in both the TAVI and control populations reflects benign implications for the incidental findings of most thrombophilias in healthy conditions. The impact of such disorders on bioprosthetic valves remains unknown. To date, only a single study has reported the prevalence of thrombophilia in patients undergoing TAVI for severe aortic stenosis. Nevertheless, the number of patients with the condition was significantly below the expected frequencies, which suggests their underdetection [5]. Although statistically underpowered, our early results do not show a higher prevalence of HALT in patients with thrombophilia. The quantitative assessment of HALT, however, shows higher thrombus volumes in patients with thrombophilia. This may reflect the incremental role in hypercoagulability once the thrombus is already formed. Following the full enrolment into this study, we will be able to evaluate the role of thrombophilia in forming HALT using a novel approach to thrombus quantification that measures volume and attenuation from CT angiography. Moreover, the post-procedural CTA will provide information on the deformation of the transcatheter bioprostheses, which has been previously shown to influence the prevalence of HALT [14].

The serial CTA will track the evolution of thrombi in patients with and without thrombophilia to understand their impact on the history of HALT. In our limited sample, the second CT angiography showed spontaneous (without starting the oral anticoagulation) resolution of HALT in the majority (four out of five) patients without thrombophilia. This suggests the futility of extensive hematologic screening of patients with HALT after TAVI. However, additional tests could be offered to patients in whom quantitative progression of HALT volume is observed between the scans despite the intensification of an antithrombotic regimen. Comprehensive quantitative thrombus characterization and bioprosthetic valve deformation in the context of the history of HALT will allow us to determine which patients should receive oral anticoagulation for this phenomenon.

## 5. Limitations

The study design has several limitations. First, self-expandable valves are overrepresented, reflecting the procedural characteristics at our center. Therefore, the results may not be fully translatable into balloon-expandable devices. Second, the patients recruited into the study were not consecutive due to the logistics necessary for additional outpatient visits in the elderly population. Finally, the study sample significantly limits the statistical power to detect differences between the groups.

## 6. Conclusions

The state-of-the-art multimodality imaging methods, along with wide hematological screening, provide valuable insights into the yet unexplored phenomenon of HALT. Recognizing the mechanisms of HALT may help prevent adverse events and prolong the durability of implanted bioprostheses.

## Figures and Tables

**Table 1:** Frequency table of thrombophilia detected in TAVI and control groups.

	TAVI (*n* = 52)	Control (*n* = 52)	*p*-Value
Any thrombophilia	8 (16%)	6 (12%)	0.565
Factor V Leiden	2 (4%)	1 (2%)	0.186
Prothrombin G20210A mutation	1 (2%)	1 (2%)	
Protein C deficiency	0 (0%)	0 (0%)	
Protein S deficiency	1 (2%)	2 (4%)	
Antithrombin deficiency	1 (2%)	1 (2%)	
Antiphospholipid syndrome	1 (2%)	0 (0%)	
Factor XI ≤ 50%	0 (0%)	0 (0%)	
Factor VIII ≥ 150%	2 (4%)	1 (2%)	

**Table 2:** Clinical characteristics and echocardiographic and procedural data of patients in the TAVI cohort.

	Thrombophilia		*p*-Value
	Yes (*n* = 8)	No (*n* = 44)	
Age, y	80 ± 6	89 ± 5	0.308
Male, *n* (%)	7 (88)	26 (59)	0.232
Body mass index, kg/m^2^	26.6 ± 5.8	25.3 ± 3.9	0.507
Hypertension, *n* (%)	5 (63)	32 (73)	0.675
Diabetes, *n* (%)	3 (38)	9 (20)	0.366
Hypercholesterolemia, *n* (%)	6 (75)	38 (86)	0.592
Coronary artery disease, *n* (%)	5 (63)	24 (55)	1.000
History of myocardial infarction, *n* (%)	2 (25)	5 (11)	0.291
Prior PCI, *n* (%)	3 (38)	10 (23)	0.396
Prior CABG, *n* (%)	1 (13)	2 (5)	0.252
History of deep vein thrombosis, *n* (%)	1 (13)	0 (0)	0.154
History of pulmonary embolism, *n* (%)	0 (0)	0 (0)	1.000
STS score, %	5.1 (1.9–8.8)	4.3 (2.4–5.7)	0.775
Bicuspid valve, *n* (%)	1 (13)	3 (7)	0.499
Echocardiographic data			
Peak aortic jet velocity, m/s	4.0 ± 1.5	4.2 ± 0.8	0.509
Mean gradient, mmHg	41 (35–54)	43 (40–52)	0.533
Maximal aortic gradient, mmHg	73 (65–87)	43 (65–82)	0.829
Aortic valve area, cm^2^	0.6 (0.4–0.7)	0.7 (0.5–0.8)	0.213
Left ventricle ejection fraction, %	55 (41–68)	61 (54–67)	0.437
Procedural data			
Transfemoral vascular access, *n* (%)	8 (100)	44 (100)	1.000
Self-expandable valve, *n* (%)	8 (100)	42 (95)	1.000
In-hospital outcomes			
Death, *n* (%)	0 (0)	0 (0)	1.000
Stroke or TIA, *n* (%)	0 (0)	0 (0)	1.000
Major vascular complication, *n* (%)	1 (13)	0 (0)	0.154
Major or life-threatening bleeding, *n* (%)	1 (13)	1 (13)	1.000
Acute kidney injury, *n* (%)	0 (0)	0 (0)	1.000

## Data Availability

Data available on request due to privacy restrictions.

## Supplementary Materials

The following supporting information can be downloaded at: https://www.mdpi.com/article/10.3390/jcdd12020062/s1, Table S1: Clinical characteristics of patients in the TAVI and control cohorts.

## References

[1] Mack M.J., Leon M.B., Thourani V.H., Makkar R., Kodali S.K., Russo M., Kapadia S.R., Malaisrie S.C., Cohen D.J., Pibarot P. (2019). Transcatheter Aortic-Valve Replacement with a Balloon-Expandable Valve in Low-Risk Patients. N. Engl. J. Med..

[2] Popma J.J., Deeb G.M., Yakubov S.J., Mumtaz M., Gada H., O’Hair D., Bajwa T., Heiser J.C., Merhi W., Kleiman N.S. (2019). Transcatheter Aortic-Valve Replacement with a Self-Expanding Valve in Low-Risk Patients. N. Engl. J. Med..

[3] Makkar R.R., Fontana G., Jilaihawi H., Chakravarty T., Kofoed K.F., De Backer O., Asch F.M., Ruiz C.E., Olsen N.T., Trento A. (2015). Possible Subclinical Leaflet Thrombosis in Bioprosthetic Aortic Valves. N. Engl. J. Med..

[4] Jilaihawi H., Asch F.M., Manasse E., Ruiz C.E., Jelnin V., Kashif M., Kawamori H., Maeno Y., Kazuno Y., Takahashi N. (2017). Systematic CT Methodology for the Evaluation of Subclinical Leaflet Thrombosis. JACC Cardiovasc. Imaging.

[5] Chakravarty T., Søndergaard L., Friedman J., De Backer O., Berman D., Kofoed K.F., Jilaihawi H., Shiota T., Abramowitz Y., Jørgensen T.H. (2017). Subclinical leaflet thrombosis in surgical and transcatheter bioprosthetic aortic valves: An observational study. Lancet.

[6] Pache G., Schoechlin S., Blanke P., Dorfs S., Jander N., Arepalli C.D., Gick M., Buettner H.J., Leipsic J., Langer M. (2016). Early hypo-attenuated leaflet thickening in balloon-expandable transcatheter aortic heart valves. Eur. Heart J..

[7] Vollema E.M., Kong W.K.F., Katsanos S., Kamperidis V., van Rosendael P.J., van der Kley F., de Weger A., Ajmone Marsan N., Delgado V., Bax J.J. (2017). Transcatheter aortic valve thrombosis: The relation between hypo-attenuated leaflet thickening, abnormal valve haemodynamics, and stroke. Eur. Heart J..

[8] Ruile P., Minners J., Breitbart P., Schoechlin S., Gick M., Pache G., Neumann F.J., Hein M. (2018). Medium-Term Follow-Up of Early Leaflet Thrombosis After Transcatheter Aortic Valve Replacement. JACC Cardiovasc. Interv..

[9] Yanagisawa R., Tanaka M., Yashima F., Arai T., Jinzaki M., Shimizu H., Fukuda K., Watanabe Y., Naganuma T., Higashimori A. (2019). Early and Late Leaflet Thrombosis After Transcatheter Aortic Valve Replacement. Circ. Cardiovasc. Interv..

[10] Rosseel L., De Backer O., Søndergaard L. (2018). Clinical valve thrombosis and subclinical leaflet thrombosis in transcatheter aortic heart valves: Clinical manifestations, diagnosis, and treatment. Precis. Clin. Med..

[11] Sondergaard L., De Backer O., Kofoed K.F., Jilaihawi H., Fuchs A., Chakravarty T., Kashif M., Kazuno Y., Kawamori H., Maeno Y. (2017). Natural history of subclinical leaflet thrombosis affecting motion in bioprosthetic aortic valves. Eur. Heart J..

[12] D’Ascenzo F., Salizzoni S., Saglietto A., Cortese M., Latib A., Franzone A., Barbanti M., Nietlispach F., Holy E.W., Burriesci G. (2019). Incidence, predictors and cerebrovascular consequences of leaflet thrombosis after transcatheter aortic valve implantation: A systematic review and meta-analysis. Eur. J. Cardiothorac. Surg..

[13] Midha P.A., Raghav V., Sharma R., Condado J.F., Okafor I.U., Rami T., Kumar G., Thourani V.H., Jilaihawi H., Babaliaros V. (2017). The Fluid Mechanics of Transcatheter Heart Valve Leaflet Thrombosis in the Neosinus. Circulation.

[14] Fukui M., Bapat V.N., Garcia S., Dworak M.W., Hashimoto G., Sato H., Gössl M., Enriquez-Sarano M., Lesser J.R., Cavalcante J.L. (2022). Deformation of Transcatheter Aortic Valve Prostheses: Implications for Hypoattenuating Leaflet Thickening and Clinical Outcomes. Circulation.

[15] Pieniak K., Jędrzejczyk S., Domaszk O., Grodecki K., Rymuza B., Huczek Z., Kochman J., Filipiak K.J., Gąsecka A. (2020). Predictors and Biomarkers of Subclinical Leaflet Thrombosis after Transcatheter Aortic Valve Implantation. J. Clin. Med..

[16] Vahanian A., Beyersdorf F., Praz F., Milojevic M., Baldus S., Bauersachs J., Capodanno D., Conradi L., De Bonis M., De Paulis R. (2022). 2021 ESC/EACTS Guidelines for the management of valvular heart disease. Eur. Heart J..

[17] Grodecki K., Olasińska-Wiśniewska A., Cyran A., Urbanowicz T., Kwieciński J., Geers J., Tamarappoo B.K., Perek B., Gocoł R., Nawara-Skipirzepa J. (2024). Quantification of Aortic Valve Fibrotic and Calcific Tissue from CTA: Prospective Comparison with Histology. Radiology.

[18] Grodecki K., Tamarappoo B.K., Huczek Z., Jedrzejczyk S., Cadet S., Kwiecinski J., Rymuza B., Parma R., Olasinska-Wisniewska A., Fijalkowska J. (2021). Non-calcific aortic tissue quantified from computed tomography angiography improves diagnosis and prognostication of patients referred for transcatheter aortic valve implantation. Eur. Heart J. Cardiovasc. Imaging.

[19] Devreese K.M.J., de Groot P.G., de Laat B., Erkan D., Favaloro E.J., Mackie I., Martinuzzo M., Ortel T.L., Pengo V., Rand J.H. (2020). Guidance from the Scientific and Standardization Committee for lupus anticoagulant/antiphospholipid antibodies of the International Society on Thrombosis and Haemostasis: Update of the guidelines for lupus anticoagulant detection and interpretation. J. Thromb. Haemost..

[20] Bursac Z., Gauss C.H., Williams D.K., Hosmer D.W. (2008). Purposeful selection of variables in logistic regression. Source Code Biol. Med..

[21] Machin D., Campbell M.J., Tan S.B., Tan S.H. (2011). Sample Size Tables for Clinical Studies.

[22] Gibson C.S., MacLennan A.H., Rudzki Z., Hague W.M., Haan E.A., Sharpe P., Priest K., Chan A., Dekker G.A. (2005). The prevalence of inherited thrombophilias in a Caucasian Australian population. Pathology.

[23] Middeldorp S. (2016). Inherited thrombophilia: A double-edged sword. Hematol. Am. Soc. Hematol. Educ. Program.

[24] Zavalloni D., Presbitero P., Lodigiani C., Mango R., Cogliati T., Quaglia I., Corrada E., Mendolicchio G.L., Gasparini G.L., Rossi M.L. (2012). Prevalence of inherited thrombophilia in patients with documented stent thrombosis. Circ. J..

