# Supplementary material for: Prothrombotic States in Transcatheter Heart Valve Leaflet Thrombosis (PROSTHESIS): Rationale and Early Results of the Observational Cohort Study
Source: J Cardiovasc Dev Dis. 2025 Feb 6;12(2):62. doi: 10.3390/jcdd12020062 (PMC11856029; doi:10.3390/jcdd12020062)
Supplement: Supplementary file 1 [file jcdd-12-00062-s001.zip › jcdd-3346385-supplementary.pdf]

Supplementary Table S1. Clinical characteristics of patients in the TAVI and control cohorts.

	**TAVI (n = 52)**	**Control (n = 52)**	**p-Value**
Age, y	79 ± 5	78 ± 5	0.399
Male, n (%)	33 (63)	33 (63)	1.00
Body mass index, kg/m^2	25.4 ± 4.0	24.5 ± 3.8	0.312
Hypertension, n (%)	37 (71)	39 (75)	0.659
Diabetes, n (%)	12 (23)	15 (29)	0.502
Hypercholesterolemia, n (%)	42 (81)	46 (88)	0.545
History of deep vein thrombosis, n (%)	1 (2)	0 (0)	0.315
History of pulmonary embolism, n (%)	0 (0)	0 (0)	1.000

Reproducibility of quantitative HALT evaluation

Interobserver agreement was measured using an intraclass correlation coefficient (ICC), with values less than 0.5 indicative of poor agreement; values between 0.5 and 0.74, moderate agreement; between 0.75 and 0.9, good agreement; and greater than 0.90, excellent agreement. Intra- and interobserver variability were measured using Bland-Altman plots with mean bias and limits of agreement.

There was excellent interobserver repeatability for thrombus volume measurement, with an ICC of 0.99 (95% CI: 0.98, 0.99; p<0.001). Additionally, Bland-Altman analysis showed that the two readers achieved a coefficient of repeatability of 29.9.9 and a mean bias of 4.8 mm3 (95% limits of agreement: −23.9 mm3, 32.0mm3) for the assessment of thrombus volume.
